# Determinants of maternal health service utilisation among refugee women in Krisan and Ampain camps, Ghana: *a cross-sectional analysis*

**DOI:** 10.3389/fgwh.2026.1807016

**Published:** 2026-09-14

**Authors:** Linus Baatiema, John Kuumuori Ganle, Francis Appiah, Daprim Samual Ogaji

**Affiliations:** 1 School of Public Health, Faculty of Public Health, University of Port Harcourt, Port-Harcourt, Nigeria; 2 Africa Centre of Excellence in Public Health & Toxicological Research (ACE-PUTOR), University of Port Harcourt, Choba, Port-Harcourt City, Rivers State, Nigeria; 3 Centre for Migration and Security, University of Business and Integrated Development Studies, Wa, Ghana; 4 Ghana Health Service, Upper West Regional Health Directorate, Wa, Ghana; 5 Department of Population, Family and Reproductive Health, School of Public Health, University of Ghana, Legon, Ghana; 6 School of Graduate Studies, Lingnan University, Tuen Mun, Hong Kong SAR, China; 7 Department of Social Sciences, Berekum College of Education, Berekum, Bono Region, Ghana

**Keywords:** antenatal care, Ghana, maternal healthcare, postnatal care, refugees, skilled delivery

## Abstract

**Background:**

Maternal healthcare utilization among refugee populations remains a major public health concern due to intersecting social, structural, and health system barriers. Guided by Andersen's Healthcare Utilization Theory (1995), this study examined the determinants of antenatal care, skilled delivery, and postnatal care among refugee women in Ghana.

**Methods:**

A cross-sectional analytical study was conducted among 410 refugee women aged 15–49 years residing in the Krisan and Ampain refugee camps in the Western Region of Ghana. Eligible women who had experienced a pregnancy within the preceding two years were recruited using a census approach. Data were collected using a structured interviewer-administered questionnaire. Because the prevalence of ANC attendance (≥4 visits) was low, Firth's penalized likelihood logistic regression was used to examine factors associated with ANC utilization. For skilled delivery and PNC utilization, conventional multivariable logistic regression was used. Adjusted odds ratios (AORs) with 95% confidence intervals (CIs) were reported, and statistical significance was assessed at *p* < 0.05. Camp location was included as a fixed effect in all models. Multilevel modeling was not employed as only two camps were included, which is insufficient for reliable random effects estimation

**Results:**

The majority of refugee women attended fewer than four ANC visits (93.9%), while 72.4% delivered with skilled birth attendants and only 31.0% received postnatal care. ANC utilization was significantly higher among women residing in Krisan Camp (AOR = 9.54; 95% CI: 1.68–54.30) and those receiving transport support (AOR = 4.31; 95% CI: 1.12–16.58). Income was not significantly associated with ANC attendance, with women earning GHS 100–499 (AOR = 0.06; 95% CI: 0.00–1.00) and ≥ GHS 500 (AOR = 0.03; 95% CI: 0.00–1.05) having lower odds of ANC attendance. Trustful social networks were paradoxically associated with lower ANC utilization (AOR = 0.32; 95% CI: 0.11–0.96). Skilled delivery was associated with education, with secondary or higher education linked to lower odds (AOR = 0.56; 95% CI: 0.31–0.99), while media exposure showed a marginal positive effect. Postnatl utilization was higher among Muslim women (AOR = 2.02; 95% CI: 1.15–3.55) and lower among married women (AOR = 0.62; 95% CI: 0.39–0.98).

**Conclusions:**

Maternal healthcare utilization among refugee women in Ghana is influenced by complex interactions between social support, access-related factors, and individual characteristics. Interventions that strengthen social networks, improve transport and service accessibility, and address contextual barriers within refugee camps are critical to improving maternal health outcomes.

## Background

Refugee women experience persistent inequities in maternal health outcomes, driven by the cumulative effects of forced displacement, social exclusion, and health system constraints (1, 2. Although global frameworks emphasise universal access to maternal healthcare, utilisation across the antenatal, intrapartum, and postnatal continuum remains uneven among refugee populations, particularly in low- and middle-income countries (LMICs) that host large numbers of displaced persons (2, 3). Increasing evidence demonstrates that maternal health service utilisation among refugees is shaped by interacting individual, social, and structural determinants rather than service availability alone (4–6).

Sub-Saharan Africa hosts a substantial and growing share of the global refugee population, accounting for more than 26% of the world's refugees and asylum seekers, with figures exceeding 9 million people as of mid-to-late 2024 (1, 2). Yet empirical evidence on maternal health service utilisation in refugee settings remains limited (7, 8). Ghana has a long-standing history of hosting refugees and has formally integrated refugee healthcare into its national health system through policy instruments such as the National Health Insurance Scheme (NHIS) and the Community-based Health Planning and Services (CHPS) programme (9, 10). Despite these inclusive policy arrangements, refugee women residing in camps continue to face significant challenges in accessing and utilising antenatal, delivery, and postnatal care services (11, 12). Existing studies from Ghana and comparable settings suggest that policy integration alone is insufficient to ensure equitable utilisation, as socioeconomic disadvantage, cultural barriers, and health system responsiveness continue to shape care-seeking behaviours among refugee women (13, 14).

Policy initiatives and humanitarian interventions in refugee-hosting contexts have largely prioritised expanding service coverage and reducing financial barriers to care **(**7, 9, 10). However, evidence suggests that such supply-side approaches often overlook critical demand-side and relational factors, including trust in providers, quality of interpersonal care, communication barriers, and women's experiences within health facilities (15–17). As a result, improvements in nominal access have not consistently translated into sustained engagement across the maternal healthcare continuum (18, 19).

Empirical evidence from humanitarian and refugee-hosting settings reveals marked variability in utilisation patterns. While some contexts report high antenatal care (ANC) attendance and facility-based delivery, these achievements frequently mask delayed initiation of care, inadequate visit frequency, and substantial out-of-pocket costs that deter continuity of care (9, 11). Studies across Africa, the Middle East, and high-income countries consistently identify language barriers, transportation challenges, limited insurance coverage, and perceived discrimination as major deterrents to timely and adequate maternal healthcare utilisation among refugee women (14, 20).

Beyond service utilisation, maternal satisfaction has emerged as a critical but under-examined determinant of continued engagement with maternal health services. Satisfaction reflects women's expectations, perceptions of fairness, interpersonal treatment, and responsiveness of care, and is strongly associated with return visits, adherence to clinical recommendations, and maternal and neonatal outcomes (18, 21). Among refugee populations, respectful and dignified care, effective communication, continuity of providers, and involvement in decision-making consistently outweigh purely technical aspects of care in shaping satisfaction (15, 16). Conversely, experiences of disrespect, language discordance, prolonged waiting times, and limited autonomy contribute to dissatisfaction and avoidance of facility-based maternal health services (19, 22).

Despite Ghana's relatively inclusive health policy environment, empirical evidence on maternal health service utilisation among refugee women remains sparse. Available studies have focused largely on access or coverage indicators, with limited examination of how socio-demographic characteristics, social cohesion, satisfaction with care, and health system responsiveness interact to influence utilisation across different refugee camp settings (8, 14, 23–25). Moreover, few studies adopt a multi-level analytical approach capable of capturing the combined influence of individual, community, and structural determinants in protracted displacement contexts.

Understanding these dynamics is essential for designing equitable, culturally responsive, and sustainable maternal health interventions. This study therefore examines the factors associated with maternal health service utilisation among refugee women in Ghana, integrating individual, social, and health system determinants to generate context-specific evidence that can inform policy, strengthen service delivery, and advance progress towards universal health coverage for displaced populations.

### Conceptual framework of maternal healthcare utilisation among refugee women in Ghana

This study is guided by Andersen's Healthcare Utilisation Theory (1995), which conceptualises healthcare use as influenced by predisposing, enabling, and need factors (26, 27). Predisposing factors, such as age, education, marital status, parity, religion, cultural beliefs, and migration history, shape refugee women's attitudes, expectations, and health-seeking behaviours (4–6). Enabling factors represent the resources and logistical supports that facilitate or constrain access to maternal healthcare, including employment, income, health insurance, social and partner support, transportation, media exposure, and proximity to health facilities (5, 9, 10). Need factors reflect perceived and evaluated health requirements that drive service utilisation, including prior obstetric experiences, perceived complications, chronic illnesses, mental health needs, and satisfaction with previous care (4, 6, 9). Applying Andersen's framework in this study allows for a systematic exploration of how these multidimensional factors interact to influence the utilisation of maternal health services antenatal care, skilled birth attendance, facility-based delivery, and postnatal care among refugee women in Ghana. By explicitly linking refugee women's socio-demographic characteristics, available resources, and health needs to service uptake, this framework provides a robust theoretical lens for identifying modifiable determinants that can inform culturally sensitive interventions, strengthen health system responsiveness, and improve maternal health outcomes in contexts of socio-economic deprivation and displacement. Its adoption is particularly relevant for this study, as it captures the complex interplay between individual, household, and structural factors that uniquely affect maternal healthcare utilisation in protracted refugee settings (Figure 1).

**Figure 1:**
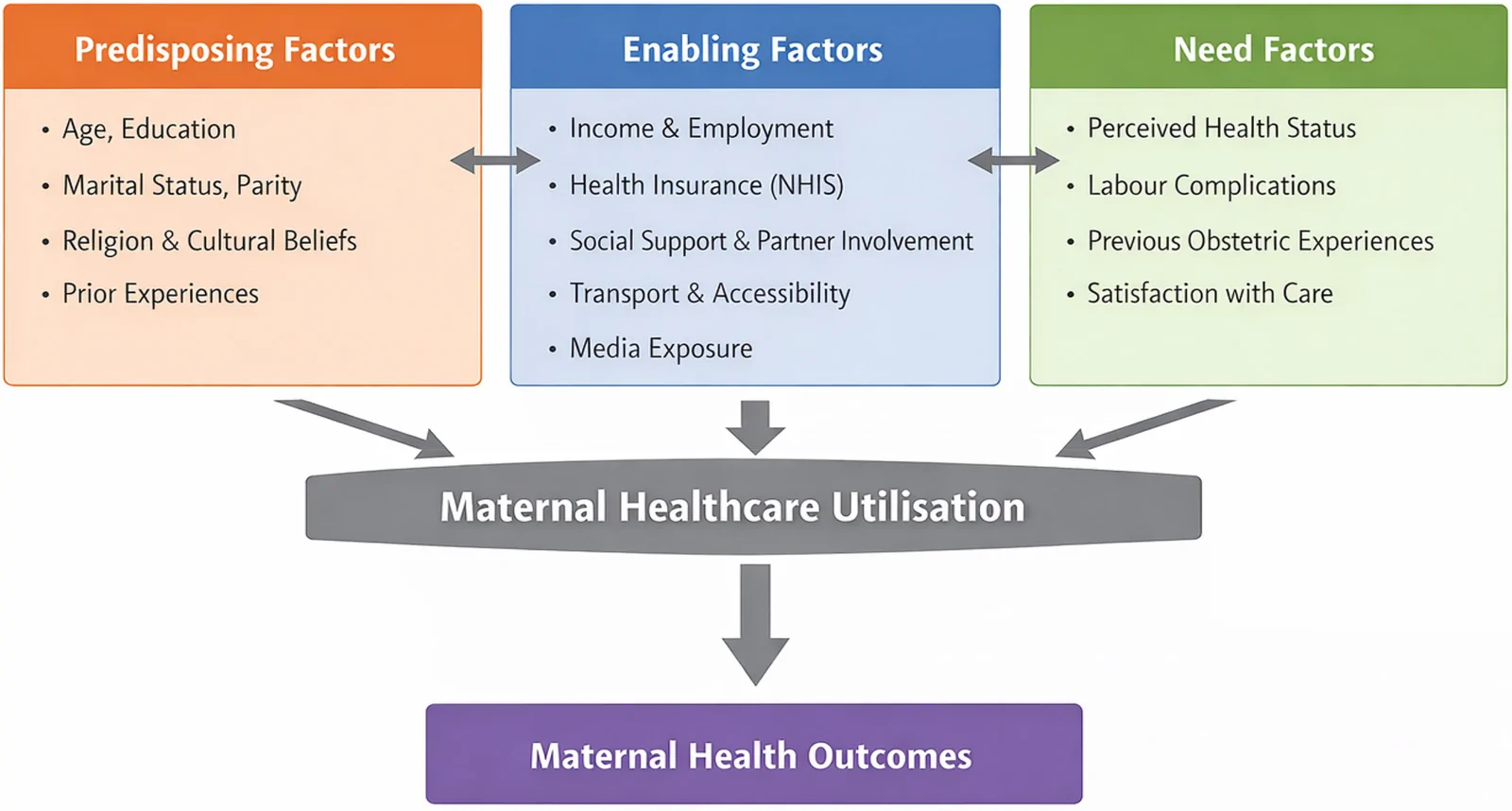
Conceptual framework nof maternal healthcare utilisation among refugee women in Ghana, adapted from Andersen's healthcare utilisation theory (26) and evidence from refugee maternal health literature (4–6, 9, 10).

## Methodology

### Study area

The study was conducted in Krisan and Ampain refugee camps in the Western Region of Ghana, which are the two currently active and officially functional refugee settlements in the country and host a diverse population of displaced persons from neighbouring African countries. Krisan camp was established in the 1990s following successive influxes of Liberian, Togolese, Sierra Leonean, Sudanese, Congolese, and other nationals and is situated in the Ellembelle District, approximately 48 km from the Ghana–Ivory Coast border; it lacks a resident health centre, requiring residents to access essential care at nearby facilities such as Saint Martin De Porres Catholic Hospital in Eikwe. Ampain camp, established in 2011 to shelter Ivorian refugees, also lies in the Ellembelle District and similarly depends on host community health centres for major healthcare needs, with referrals to hospitals located some distance away. estimated 1,250 women of reproductive age in both camps, 578 were mothers, with 361 (46.2%) residing in Krisan Camp and 217 (17.4%) in Ampain Camp. According to Baatiema, Ogaji, and Ganle (20), refugee women in these camps encounter multiple barriers to maternal healthcare, including language challenges, financial constraints, transportation difficulties, and negative provider attitudes, underscoring the complexity of care-seeking in these settings. Both camps are managed under the coordination of the Ghana Refugee Board and the United Nations High Commissioner for Refugees (UNHCR), with basic services such as education, security, and maternal health integrated into the national system while economic activities remain predominantly information (Figure 2).

**Figure 2:**
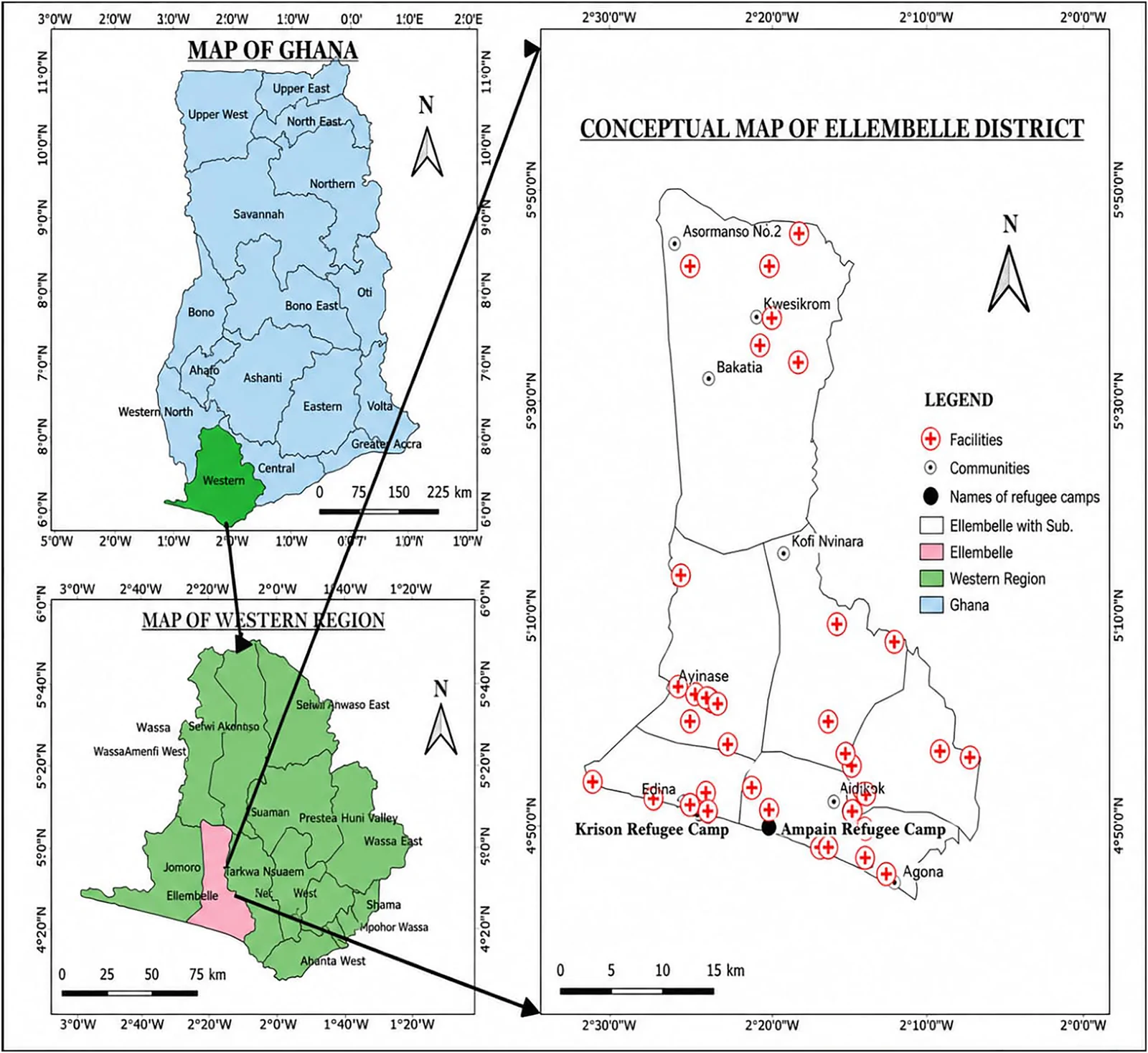
Map of ellembelle district showing the two refugee camps. *Source:* Author's own construction (2025).

### Study design

This study employed a cross-sectional analytical design to examine factors associated with maternal health service utilisation among refugee women in Ghana. A Community-Based Analytical cross-sectional approach was chosen because it allows for the simultaneous assessment of multiple determinants socio-demographic, cultural, and health system factors at a single point in time, providing a snapshot of utilisation patterns and their correlates. The design is appropriate for identifying associations between maternal health service utilisation and potential predictors such as age, education, marital status, parity, social support, and satisfaction with care. Data were collected from women of reproductive age residing in the Krisan and Ampain refugee camps, enabling comparisons across camps and identification of context-specific barriers and facilitators to maternal healthcare.

### Study population

The study population comprised refugee women of reproductive age (15–49 years) residing in the Krisan and Ampain refugee camps in the Western Region of Ghana. These camps are the only functional refugee settlements in the country and host a diverse population of women from different cultural and socio-economic backgrounds. Eligible participants were women who had been residents of the camps for at least six months and had experienced a pregnancy within the last two years, ensuring relevance to recent maternal healthcare utilisation. Women who were critically ill or unable to provide informed consent were excluded.

### Sampling method and recruitment process

Given the relatively small size of the target population, a census approach was employed to maximize coverage and ensure representativeness. All eligible women in the Krisan and Ampain refugee camps were approached for participation. Trained research assistants visited every household within the camps, provided a detailed explanation of the study objectives, and obtained informed consent prior to data collection. Eligibility criteria included being a resident of the camp for at least six months and having experienced a pregnancy within the previous two years. Women who were critically ill or unable to provide consent were excluded. Of the estimated 578 eligible mothers (361 in Krisan Camp and 217 in Ampain Camp), 410 women consented to participate. The remaining eligible women were either unavailable during the data collection period or declined participation, often due to the perception that participation would not provide financial benefit. This approach ensured a high degree of representativeness while accounting for practical constraints in accessing the full population.

### Data collection

Data were collected using a structured, interviewer-administered questionnaire adapted from validated instruments in previous refugee maternal health studies (4, 6, 10). The questionnaire captured information on socio-demographic characteristics (age, education, employment, income, marital status), maternal health service utilisation (antenatal care, facility delivery, postnatal care), maternal satisfaction with healthcare services, social support and community cohesion, and health system factors including access, transportation, insurance coverage, and provider continuity. Data collectors were trained on ethical research practices, cultural sensitivity, and standardised administration procedures. Data were collected electronically using CSPro (Census and Survey Processing System), which allowed real-time data entry, validation checks, and efficient management of survey responses. The questionnaire was pretested among a population of Liberian refugee women residing in the former Buduburam settlement (Central Region, Ghana), a population with similar socio-demographic and displacement characteristics to the study population. This site was chosen because it is no longer an active camp but still hosts displaced women with comparable maternal health experiences. Data collection started on 18th October 2024 and ended at 20th December 2024. each survey lasted for about 15 min.

### Study variables

The primary dependent variable for this study were three separate indicators of maternal health service utilization; (1) attendance of at least four antenatal care visits (yes/no), (2) skilled delivery (yes/no), and (3) postnatal care attendance within six weeks of delivery (yes/no). Each indicator was analysed as a distinct outcome in separate multivariable logistic regression models.

The independent variables encompassed socio-demographic, social, health system, and maternal satisfaction factors. Socio-demographic variables included age, education, employment, income, marital status, and parity. Social factors captured family support, community cohesion, and trustful networks. Health system factors comprised access to services, transportation availability, cost, continuity of care, and the provision of culturally appropriate services. Maternal satisfaction indicators included respectful treatment by healthcare providers, involvement in decision-making, waiting times, and perceived fairness of care. These variables were selected to comprehensively capture individual, social, and structural determinants of maternal health service utilisation among refugee women.

### Data analysis

Data were entered and managed using CSPro version 7.6 and exported to STATA version 18 for statistical analysis. Analysis was conducted in stages. First, descriptive statistics, including frequencies, percentages, means, and standard deviations, were used to summarise respondents' socio-demographic characteristics and patterns of maternal health service utilisation, including antenatal care attendance, facility-based delivery, and postnatal care.

Second, maternal healthcare service utilization was examined using multivariable regression analyses. Because the prevalence of ANC attendance (≥4 visits) was low, Firth's penalized likelihood logistic regression was used to examine factors associated with ANC utilization. This approach reduces small-sample bias and provides more reliable estimates for rare outcomes (Puhr, et al, (38)). For skilled delivery and postnatal care (PNC) utilisation, conventional multivariable logistic regression was used because the outcomes had sufficient numbers of events for maximum likelihood estimation. Adjusted odds ratios (AORs) with 95% confidence intervals (CIs) were reported, and statistical significance was assessed at *p* < 0.05.

Although the data have a hierarchical structure (women nested within camps), multilevel modeling was not employed because only two camps (Krisan and Ampain) were included in the study. Methodological simulation studies suggest that reliable estimation of random effects and cluster-level variance requires a minimum of 20–30 clusters (level 2 units) (28, 29). With only two camps, a multilevel model would be underpowered and produce unreliable variance estimates. Therefore, ordinary multivariable logistic regression with robust standard errors was used, and camp location (Krisan vs. Ampain) was included as a fixed effect (independent variable) in all regression models to adjust for camp-level differences. Findings were presented in tables with narrative explanations to highlight key determinants of maternal healthcare utilisation among refugee women in the study camps.

## Results

### Socio-demographic characteristics and maternal health service utilisation of study participants

The study involved 410 refugee women from Ampain (78.10%) and Krisan (22.00%) camps. The mean age of participants was 32.40 years (standard deviation: 8.70 years; range: 15–49 years). Most participants were aged 35–39 years (24.60%) and had completed secondary (46.30%) or higher education (36.60%). Living arrangements included partners (34.20%), family (31.20%), and children (28.80%). Over half (52.90%) reported trustful social networks. Employment varied, with 28.80% unemployed and 67.10% earning GHS 100–499 monthly. Most women were married (52.00%) and Christian (88.10%). Parity ranged from 1 to 2 children (49.50%) to ≥5 children (9.50%). Media exposure was highest for radio (77.80%). Access barriers included language (38.8%) and transportation (39.80%), while partner involvement in maternal care was low (ANC 10.70%, delivery 10.20%, PNC 5.40%). Knowledge of ANC and PNC was predominantly moderate to high. Labour complications were reported by 16.30%. Maternal health services were available to 62.70% of women; 67.60% paid for care, and 84.90% experienced difficulty accessing services. Caesarean deliveries accounted for 16.10% of births. Satisfaction with care was high: ANC and PNC (97.30%) and delivery (95.90%), reflecting positive experiences among those accessing services. (Table 1).

**Table 1:** Socio-Demographic characteristics and maternal health service utilisation of study participants.

Variables	Frequency (*N*)	Percentage (%)
Predisposing factors		
Age group (years)		
15–19	41	10.00
20–24	71	17.30
25–29	82	20.0
30–34	62	15.10
35–39	101	24.60
40–44	31	7.60
45–49	22	5.40
Education level		
Primary	70	17.10
Secondary	190	46.30
Higher	150	36.60
Marital Status		
Single	97	23.70
Separated/Divorced	52	12.7
Married	213	52.00
Other	48	11.700
Parity		
1–2 births	203	49.50
3–4 births	168	41.00
≥5 births	39	9.50
ANC Knowledge		
Low	50	12.20
Moderate	141	34.40
High	219	53.40
Religion		
Christian	361	88.10
Muslim	42	10.20
Other	7	1.70
Enabling factors		
Employment Status		
Unemployed	118	28.80
Homemaker	20	4.90
Employed	49	12.00
Student	58	14.20
Other	165	40.20
Monthly income (GHS)		
0	14	3.41
1–99	66	16.10
100–499	275	67.10
≥500	55	13.40
Living Arrangement		
With children	118	28.80
Friend	8	2.00
In-law	9	2.20
Alone	7	1.70
Partner	140	34.20
Family	128	31.20
Trustful Social Network		
No	193	47.10
Yes	217	52.90
Receives financial support (gift)		
No	106	25.90
Yes	304	74.10
Husband/partner ANC support		
No	366	89.30
Yes	44	10.70
Partner involved in PNC		
No	388	94.60
Yes	22	5.40
Reads newspaper/magazine		
No	252	61.50
Yes	158	38.50
Listens to radio		
No	91	22.20
Yes	319	77.80
Watches television		
No	222	54.20
Yes	188	45.90
Lack of money for transport/care		
No	247	60.20
Yes	163	39.80
Pays for maternal health services		
No	133	32.40
Yes	277	67.60
Difficulty accessing MHS		
No	62	15.10
Yes	348	84.90
Need factors		
Labour/delivery complications		
No	343	83.70
Yes	67	16.30
Camp location		
Ampain	320	78.1
Krisan	90	22.0

Source: Field Survey, 2024.

### Utilization of ANC service, institutional delivery and PNC among refugee women at Krisan and Ampain refugee camp in Ghana

Maternal health service utilisation among refugee women in Krisan and Ampain camps showed considerable variation across the continuum of care. Antenatal care (ANC) attendance was low, with only 25 women (6.10%) completing four or more visits, while the majority, 385 women (93.90%), attended fewer than four ANC visits. Facility-based delivery was higher, with 304 women (74.20%) delivering in a health facility compared to 106 women (25.90%) who delivered outside health facilities. Similarly, skilled birth attendance was received by 297 women (72.40%), while 113 women (27.60%) did not receive skilled care during childbirth. Postnatal care (PNC) utilisation was relatively limited, with only 127 women (31.00%) attending PNC services within six weeks of delivery, whereas 283 women (69.00%) did not access postnatal services. Overall, these findings indicate gaps in the continuity of maternal healthcare utilisation, particularly for ANC and PNC, underscoring the need for interventions that promote consistent engagement with maternal health services (Table 2).

**Table 2:** Utilization of ANC Service, institutional delivery and PNC Among Refugee Women at Krisan and Ampain Refugee Camp in Ghana.

Category	Frequency	Percentage
ANC visits		
Less than 4	385	93.90
4 or more	25	6.10
Skilled Birth		
No	113	27.60
Yes	297	72.40
PNC		
No	283	69.00
Yes	127	31.00
Total	410	100

Source: Field Survey, 2024.

### Factors associated with maternal healthcare service utilization

Table 3 presents the multivariable regression analyses examining the associations between predisposing, enabling, and need factors and maternal healthcare service utilisation. Multivariable logistic regression was used for the skilled delivery and postnatal care (PNC) models, whereas Firth's penalized logistic regression was used for the antenatal care (ANC). For ANC utilisation, income was not significantly associated with attendance. Although women earning GHS 100–499 (Adjusted OR = 0.06; 95% CI: 0.00–1.00; *p* = 0.050) and those earning ≥GHS 500 (Adjusted OR = 0.03; 95% CI: 0.00–1.05; *p* = 0.047) had lower odds of ANC attendance compared with women with no income, these associations were not statistically significant. Trustful social networks significantly decreased the likelihood of ANC attendance (Adjusted OR = 0.32; 95% CI: 0.11–0.96; *p* = 0.042). Women reporting money for transport (Adjusted OR = 4.31; 95% CI: 1.12–16.58; *p* = 0.034) and camp location (Krisan) (Adjusted OR = 9.54; 95% CI: 1.68–54.30; *p* = 0.011) were significantly associated with higher ANC utilization. Other variables, including age, education, marital status, parity, ANC knowledge, religion, employment, living arrangement, receiving gifts, husband ANC support, media exposure, were not statistically significant.

**Table 3:** Factors associated with maternal healthcare service utilization.

Variable	ANC adjusted OR (95% CI)	ANC *p*-value	Skilled delivery adjusted OR (95% CI)	Skilled delivery *p*-value	PNC adjusted OR (95% CI)	PNC *p*-value
Predisposing Factors						
Age Group						
Ref: 15–19	**1**	–	**1**	–	**1**	–
20–24	2.51 (0.26–23.91)	0.43	0.85 (0.30–2.42)	0.76	2.34 (0.59–9.23)	0.23
25–29	3.01 (0.19–47.44)	0.43	1.14 (0.35–3.77)	0.83	3.14 (0.70–14.17)	0.14
30–34	3.26 (0.18–58.76)	0.42	1.51 (0.39–5.91)	0.56	2.12 (0.38–11.79)	0.39
35–39	1.08 (0.05–22.32)	0.96	1.22 (0.32–4.66)	0.77	1.60 (0.27–9.40)	0.60
40–44	0.39 (0.01–17.64)	0.63	4.56 (0.74–28.06)	0.10	3.02 (0.33–27.56)	0.33
45–49	3.49 (0.12–99.48)	0.47	1.96 (0.37–10.42)	0.43	4.94 (0.51–47.77)	0.17
Education						
Ref: Primary	1		1		1	
Secondary	1.76 (0.43–7.22)	0.43	0.32 (0.13–0.76)	**0** **.** **01**	2.19 (0.84–5.69)	0.11
Higher	2.01 (0.43–9.45)	0.38	0.27 (0.11–0.69)	**0** **.** **01**	1.19 (0.43–3.29)	0.74
Marital Status						
Ref: Single	1		1		1	
Separated/Divorced	3.07 (0.71–13.25)	0.13	1.30 (0.52–3.25)	0.57	0.66 (0.19–2.36)	0.52
Married	0.91 (0.20–4.04)	0.90	1.20 (0.51–2.82)	0.68	0.33 (0.11–0.98)	**0** **.** **04**
Other	1.08 (0.17–6.83)	0.94	0.89 (0.35–2.27)	0.80	0.83 (0.26–2.66)	0.75
Parity						
Ref: 1–2 births	1		1		1	
3–4 births	0.55 (0.16–1.85)	0.33	1.21 (0.66–2.20)	0.54	0.76 (0.36–1.59)	0.46
≥5 births	0.68 (0.06–7.35)	0.75	0.80 (0.24–2.69)	0.72	1.41 (0.27–7.28)	0.68
ANC Knowledge						
Ref: Low	1		1		1	
Moderate	0.36 (0.08–1.69)	0.20	0.79 (0.36–1.73)	0.56	1.53 (0.58–4.06)	0.40
High	0.92 (0.25–3.45)	0.90	1.01 (0.48–2.13)	0.99	1.15 (0.45–2.95)	0.76
Religion						
Ref: Christian	1		1		1	
Muslim	0.12 (0.01–4.40)	0.17	4.44 (0.91–21.76)	0.07	11.37 (1.47–87.69)	**0** **.** **02**
Other	1.54 (0.04–57.50)	0.82	1.42 (0.20–9.88)	0.73	2.35 (0.10–53.59)	0.59
Enabling Factors						
Employment						
Ref: Unemployed	1		1		1	
Homemaker	7.49 (0.34–163.28)	0.20	1.07 (0.19–6.02)	0.94	3.23 (0.33–31.42)	0.31
Employed	1.27 (0.18–9.07)	0.81	2.41 (0.84–6.94)	0.10	2.87 (0.78–10.61)	0.11
Student	1.07 (0.11–10.23)	0.95	1.13 (0.43–2.97)	0.80	1.54 (0.45–5.27)	0.49
Other	1.86 (0.39–8.78)	0.43	1.10 (0.54–2.24)	0.80-	1.38 (0.58–3.27)	0.47
Income (GHS)						
Ref: 0.00	1		1		1	
1–99	0.50 (0.04–6.32)	0.59	0.83 (0.15–4.56)	0.84	0.76 (0.09–6.79)	0.81
100–499	0.06 (0.00–1.00)	**0** **.** **05**	1.50 (0.26–8.76)	0.65	0.32 (0.03–3.24)	0.34
≥500	0.03 (0.00–1.05)	**0** **.** **05**	2.30 (0.30–17.90)	0.43	0.21 (0.02–2.91)	0.25
Living Arrangement						
Ref: With children	1		1		1	
Friend	2.44 (0.19–30.59)	0.49	0.44 (0.07–2.58)	0.36	0.07 (0.01–1.01)	0.05
In-law	0.15 (0.00–7.72)	0.33	2.41 (0.32–18.44)	0.40	-	-
Alone	0.25 (0.01–12.72)	0.49	0.38 (0.06–2.35)	0.30	0.10 (0.01–1.58)	0.10
Partner	0.65 (0.15–2.78)	0.56	0.86 (0.41–1.82)	0.69	0.66 (0.26–1.65)	0.37
Family	1.09 (0.24–4.93)	0.91	1.80 (0.74–4.36)	0.19	0.35 (0.12–1.03)	0.06
Trustful Network						
Ref: No	1		1		1	
Yes	**0.32** **(0.11–0.96)**	**0** **.** **04**	1.03 (0.60–1.79)	0.91	1.14 (0.54–2.42)	0.73
Receives Gift						
Ref: No	1		1		1	
Yes	2.47 (0.61–10.00)	0.21	0.66 (0.29–1.51)	0.33	1.17 (0.40–3.45)	0.77
Husband ANC Support						
Ref: No	1		1		1	
Yes	0.41 (0.08–2.13)	0.29	1.14 (0.53–2.49)	0.73	1.13 (0.43–2.98)	0.81
Partner in PNC						
Ref: No	1		1		1	
Yes	-	-	0.53 (0.20–1.43)	0.21	-	-
Reads News						
Ref: No	1		1		1	
Yes	1.36 (0.55–3.38)	0.51	0.62 (0.38–1.00)	**0** **.** **05**	0.59 (0.31–1.13)	0.11
Listens to Radio						
Ref: No	1		1		1	
Yes	1.84 (0.57–5.96)	0.31	1.16 (0.65–2.05)	0.62	1.97 (0.91–4.27)	0.09
Watches TV						
Ref: No	1		1		1	
Yes	1.10 (0.31–3.98)	0.88	0.94 (0.50–1.76)	0.85	1.38 (0.61–3.13)	0.44
Money for Transport						
Ref: No	1		1		1	
Yes	**4.31** **(1.12–16.58)**	**0** **.** **03**	1.14 (0.58–2.22)	0.70	1.04 (0.44–2.46)	0.93
Pays for Services						
Ref: No	1		1		1	
Yes	0.88 (0.33–2.32)	0.80	1.13 (0.67–1.90)	0.65	-	-
Difficulty Accessing MHS						
Ref: No	1		1		1	
Yes	0.36 (0.11–1.22)	0.10	0.74 (0.35–1.54)	0.42	0.66 (0.27–1.62)	0.36
Language Barrier						
Ref: No	1		1		1	
Yes	2.49 (0.54–11.58)	0.24	1.72 (0.86–3.44)	0.13	1.56 (0.64–3.83)	0.33
Need Factors						
Labor Complications						
Ref: No	1		1		1	
Yes	1.99 (0.03–129.30)	0.75	0.78 (0.40–1.52)	0.47	0.76 (0.30–1.91)	0.56
Camp Location						
Ref: Ampain	1		1		1	
Krisan	**9.54** **(1.68–54.30)**	**0** **.** **01**	0.44 (0.17–1.15)	0.09	0.75 (0.19–2.93)	0.68

Bold values indicate statistically significant associations at *p* < 0.05. Reference category for each variable is denoted by “Ref.” ANC, Antenatal Care; PNC, Postnatal Care; MHS, Maternal Health Services; GHS, Ghana Cedis. Odds ratios (OR) and 95% confidence intervals (CI) are presented for each variable. Source: Field Survey, 2024.

Regarding PNC utilization, marital status and religion were significant predictors. Married women had lower odds of PNC attendance compared with single women (Adjusted OR = 0.33; 95% CI: 0.11–0.98; *p* = 0.047), while Muslim women had significantly higher odds compared with Christians (Adjusted OR = 11.37; 95% CI: 1.47–87.69; *p* = 0.02). Living arrangement and listening to the radio were marginally associated with PNC utilization, while other predisposing and enabling factors did not show significant associations (Table 3).

## Discussion

This study explored maternal health service utilization and its associated factors among refugee women living in the Krisan and Ampain refugee camps in Ghana, focusing on antenatal care (ANC), skilled delivery, and postnatal care (PNC). The findings provide insights into how various socio-demographic, economic, and contextual factors influence the uptake of these essential services in humanitarian settings. While several results confirm patterns reported in existing literature from low- and middle-income countries, others differ, reflecting the distinct realities of displaced populations. The following discussion unpacks these findings in relation to relevant studies, drawing attention to both supporting and contradicting evidence from the broader literature.

### Factors associated with ANC uptake

This study found that antenatal care (ANC) utilization among refugee women was significantly influenced by multiple factors, including income, trustful social networks, access to transport support, and camp location. Income was not significantly associated with antenatal care (ANC) utilization among refugee women in this study. Although women earning GHS 100–499 and ≥GHS 500 had lower adjusted odds of ANC utilization than women with no income, these differences were not statistically significant and should therefore not be interpreted as evidence of an association. According to Andersen's Healthcare Utilization Theory, income is an enabling factor that may influence access to healthcare services. Previous studies among refugee populations have reported that financial constraints and out-of-pocket costs are important barriers to ANC utilization (9, 13). For example, Tappis et al. (9) found that maternal healthcare costs limited care-seeking among refugees in Jordan and Lebanon, while Moudi et al. (13) identified financial constraints and lack of health insurance as barriers to ANC uptake among Afghan refugee women. However, unlike these studies, our findings did not demonstrate a statistically significant relationship between income and ANC utilization. This suggests that, within the Krisan and Ampain refugee camps, factors other than individual income may have played a more prominent role in influencing ANC utilization, possibly due to the availability of humanitarian assistance and subsidized maternal health services. Further research is needed to better understand the relative contribution of socioeconomic factors to maternal healthcare utilization in refugee settings.

ANC utilization was also significantly lower among women with trustful social networks. Guided by Andersen's framework, social networks serve as an enabling factor, typically facilitating care-seeking through emotional support, health information, and practical assistance. This finding, however, is unexpected and contrasts with studies among refugee populations in Europe and Australia, where supportive community relationships enhanced engagement with antenatal services (30, 31). The negative association observed in our study may be explained by the nature of trust within the camp setting it is plausible that women with trusting networks rely more on informal sources of support, such as traditional birth attendants or community advice, rather than formal health services. Similar findings were reported by Moudi et al. (13), where 20.45% of Afghan refugee women relied on traditional birth attendants despite having community connections. Alternatively, trustful networks may reinforce existing cultural norms that discourage formal ANC attendance, or women may share negative experiences and misconceptions about the quality of care, leading to avoidance of health facilities. This finding implies that while social networks are generally considered beneficial, their influence is context-dependent, and ANC promotion strategies must engage community leaders to ensure that social networks transmit accurate health information and encourage formal care-seeking.

Access to transport support was a significant determinant of ANC use, with women reporting money for transport having higher odds of ANC utilization. Within Andersen's framework, transport functions as an enabling factor that reduces physical barriers to care. From the Three Delays Model perspective, transport availability addresses the second delay the delay in reaching a health facility. This finding is strongly supported by evidence from refugee settings, where transportation availability, including transport vouchers or mobile outreach services, significantly increases ANC attendance (4, 5, 9). Nakisita et al. (32) similarly identified logistical barriers, including transport, as key constraints to ANC completion among urban refugee adolescents in Kampala. Conversely, in better-resourced urban refugee contexts, transport barriers are less critical, and ANC utilization is influenced more by individual factors such as health literacy (10). The implication is that improving transport support through vouchers, community transport schemes, or mobile clinics is essential for facilitating timely and consistent ANC attendance among refugee women.

Camp location was strongly associated with ANC attendance, with women residing in Krisan camp having significantly higher odds of utilization compared with those in Ampain camp. This finding aligns with evidence from Ghana and other refugee-hosting contexts showing that proximity to health facilities, the presence of UNHCR-supported services, and regular outreach activities improve maternal healthcare utilization (2, 9). Camps with strong humanitarian–government collaborations reduce logistical and financial barriers, facilitating repeated ANC visits. Orikiriza et al. (11) observed that refugee mothers in Uganda were slightly less likely to receive ANC than host populations but more likely to deliver in health facilities, highlighting the camp-specific variation in service access. The difference between Krisan and Ampain camps may reflect disparities in health facility proximity, availability of skilled providers, intensity of outreach services, and the functional integration of camp health systems with national health structures. This finding implies that well-coordinated service delivery and sustained outreach in camps are essential for improving ANC uptake, and that resource allocation should be tailored to the specific needs of each camp setting. Interestingly, marital status which was significant in the original analysis was not statistically significant in the updated model, suggesting that the factors identified above may be more robust predictors of ANC utilization in this population.

### Factors associated with skilled delivery

This study found that skilled delivery was significantly associated with women's education, with those having secondary or higher education showing lower odds of facility-based childbirth, while reading newspapers or magazines had a marginal protective effect. According to Andersen's Healthcare Utilization Theory, education is a predisposing factor that typically increases knowledge, awareness, and positive health-seeking behaviors. However, the inverse relationship observed in this study suggests that structural and contextual barriers may outweigh educational advantages in refugee settings. Educated women may prefer home births due to cultural norms, mistrust of health facilities, prior negative experiences, or fear of surgical interventions such as cesarean sections (14, 33, 34). From the perspective of the Three Delays Model, these barriers relate to the second delay (delay in reaching a facility) and the third delay (delay in receiving adequate care), highlighting that knowledge alone does not guarantee utilization if enabling factors such as transport, facility accessibility, and quality of care are inadequate.

Reading newspapers or magazines showed a marginal protective effect on skilled delivery, representing an enabling factor within Andersen's framework, as media exposure increases awareness about facility-based births, danger signs during labor, and the benefits of skilled attendance. This aligns with evidence that health information via media improves maternal health service utilization (9, 10). In terms of the Three Delays Model, media exposure primarily addresses the first delay (deciding to seek care) by influencing knowledge, attitudes, and motivation to access health services. The marginal effect suggests that while information can encourage skilled delivery, structural and socio-cultural barriers still play a decisive role in preventing full utilization. These findings imply that interventions aimed at increasing skilled delivery should combine education and awareness campaigns with practical support, including transport facilitation, quality improvement of maternal services, and community engagement to reduce home-birth preferences.

### Factors associated with postnatal care (PNC) uptake

The higher odds of PNC attendance among Muslim women may reflect the supportive role of religious communities in encouraging maternal healthcare utilization. Social networks within religious groups can provide emotional support, practical assistance such as accompaniment to health facilities, and reinforcement of positive health behaviors, which function as enabling factors in Andersen's model (4, 35, 36). These findings align with evidence from refugee populations in Nordic countries, where social cohesion and trust in community networks significantly facilitated maternal health service use (10, 37).

In contrast, married women were less likely to utilize PNC services. Marital status influences household power dynamics, autonomy, and competing domestic responsibilities, which may constrain women's ability to seek care (4, 35). Similar patterns have been observed among urban refugee adolescents in Uganda, where women living with partners often had lower autonomy in health decisions, resulting in delayed maternal service utilization (34). In the framework of the Three Delays Model, these dynamics contribute to the first delay, as women may require spousal approval before attending PNC, and potentially to the second delay if domestic obligations limit mobility. This contrasts with studies from Somalia and Jordan, where partner support was positively associated with PNC attendance, indicating that the influence of marital status is highly context-specific (9, 33, 34).

Several factors in this study, while not statistically significant, showed tendencies suggesting subtle influences on maternal healthcare utilization. For example, listening to the radio and exposure to newspapers or magazines appeared to slightly increase the likelihood of ANC and skilled delivery, while supportive living arrangements hinted at modest improvements in postnatal care attendance. Guided by Andersen's Healthcare Utilization Theory, these factors can be viewed as enabling resources, providing access to health information, awareness of service benefits, and social support that may encourage women to seek care (9, 10). Similarly, social support from extended families or communal living arrangements, though not significant, aligns with evidence from refugee populations in Uganda and Ghana showing that household and community support facilitates maternal service use by assisting with childcare, transport, or decision-making (2, 11). From the perspective of the Three Delays Model, these trends likely influence the first delay (decision to seek care) by reinforcing positive health behaviors and the second delay (reaching care) by helping women overcome logistical barriers. Even though these associations did not reach statistical significance, they underscore the importance of social and informational enablers, suggesting that interventions targeting media-based health education and household, or community support could subtly improve maternal healthcare uptake among refugee women (4, 5).

### Strengths and limitations

This study provides original evidence on maternal healthcare utilization among refugee women in Ghana, a population often underrepresented in research. Guided by Andersen's Healthcare Utilization Theory, the study systematically examined predisposing, enabling, and need-based factors influencing antenatal care, skilled delivery, and postnatal care. The use of multivariable logistic regression, adjusting for confounders, strengthens the reliability of the reported associations, and the inclusion of multiple maternal healthcare outcomes offers a comprehensive view of service utilization across the continuum of care. Additionally, the sample was drawn from both established and remote refugee settlements, enhancing the generalizability of the findings to similar contexts. Nevertheless, several limitations should be acknowledged. First, the cross-sectional design limits causal inference, preventing determination of temporality between determinants and healthcare utilization. Second, data were self-reported, which may introduce recall or social desirability bias. Third, some influential variables, such as cultural beliefs, prior obstetric complications, and quality of care, were not measured. Fourth, the study focused only on refugee women aged 15–49, potentially limiting generalizability to other age groups or male partners who influence care decisions. Fifth, certain subgroups, including newly arrived or highly transient refugees, may have been underrepresented. Sixth, the study included only two refugee camps (Krisan and Ampain); although the data have a natural hierarchical structure, multilevel modeling was not employed because reliable estimation of random effects and cluster-level variance requires a minimum of 20–30 clusters. With only two camps, ordinary multivariable logistic regression with camp location included as a fixed effect was used instead. Seventh, the study did not collect data on distance, travel time, or cost to health facilities, which may influence maternal health service utilisation. Eighth, all refugee women in the study camps are universally covered by the National Health Insurance Scheme (NHIS) through support from UNHCR and the Ghana Refugee Board, and they receive monthly stipends; consequently, NHIS coverage showed no variation (100%) and could not be included as a predictor, and findings may not generalise to refugee populations without universal health insurance coverage.

### Implications for policy and practice

Improving maternal healthcare utilization among refugee women requires coordinated, multi-level interventions. Strengthening community and religious networks can provide practical, emotional, and informational support, helping women make timely decisions to seek care and navigate barriers to service access. Health promotion campaigns delivered through culturally appropriate media, including radio, newspapers, and community outreach, can raise awareness about the importance of antenatal care, skilled delivery, and postnatal care, reinforcing positive health behaviors and dispelling misconceptions. Structural interventions are also critical; transport support, mobile clinics, and outreach services can reduce logistical and financial obstacles, particularly for women in camps and remote settlements who face challenges in reaching health facilities. Household and marital dynamics should be addressed by empowering women to make autonomous health decisions, while engaging partners and families to create supportive home environments that facilitate maternal healthcare attendance. At the policy level, these strategies should be integrated into national refugee health programs and coordinated across health authorities, humanitarian organizations, and local stakeholders to ensure equitable access, continuity of care, and reductions in maternal morbidity and mortality. Ultimately, these measures can create an enabling environment where refugee women are informed, supported, and able to utilize the full continuum of maternal healthcare services, thereby improving maternal and neonatal outcomes in vulnerable populations.

## Conclusion

This study highlights the complex interplay of social, structural, and individual factors influencing maternal healthcare utilization among refugee women in Ghana. ANC utilization was significantly influenced by income, trustful social networks, transport support, and camp location; income was not significantly associated with ANC attendance, trustful social networks were paradoxically associated with lower ANC uptake, while transport support and residence in Krisan camp significantly enhanced utilization. For skilled delivery, higher education was associated with lower odds of facility-based childbirth, and media exposure showed a marginal protective effect, while for PNC, Muslim women had higher attendance and married women were less likely to utilize services. The findings underscore the importance of targeted interventions that strengthen community support, enhance health education, improve logistical access, and empower women to make autonomous health decisions. Integrating these strategies into national refugee health programs, in collaboration with humanitarian organizations, is critical for ensuring equitable access, continuity of care, and reductions in maternal morbidity and mortality. Addressing both social and systemic determinants of care can promote the full continuum of maternal health services and improve health outcomes for this vulnerable population.

## Data Availability

The original contributions presented in the study are included in the article/Supplementary Material, further inquiries can be directed to the corresponding author.
